# The Volume-Outcome Relationship and Traveling for Hepatobiliary and Pancreatic Surgery: A Quantitative Analysis of Patient Perspectives

**DOI:** 10.7759/cureus.11023

**Published:** 2020-10-18

**Authors:** Jesse Chou, Vishal Somnay, Alyssa Woodwyk, Gitonga Munene

**Affiliations:** 1 Surgery, Western Michigan University Homer Stryker M.D. School of Medicine, Kalamazoo, USA; 2 Radiology, Western Michigan University Homer Stryker M.D. School of Medicine, Kalamazoo, USA; 3 Biostatistics, Western Michigan University Homer Stryker M.D. School of Medicine, Kalamazoo, USA

**Keywords:** health policy making, volume-outcomes, decision-making

## Abstract

Despite the well-established relationship between volume and outcomes, patients continue to have procedures performed at low-volume hospitals. The factors patients use to make the complex decision of where to have hepatopancreaticobiliary (HPB) surgery remain poorly characterized. A novel survey instrument was administered to all patients who had undergone HPB surgery at two university-affiliated community hospitals. 76 patients participated in the study (89% response rate). The majority of patients were unaware of the volume-outcome relationship (58.8%). No demographic factors differed between patients who were or were not aware except for patient research. Physician factors were the most important selection category (64.4%). Only 28.9% of patients were willing to travel more than two hours to have an operation performed at a hospital with a high volume/improved quality. Despite many voices calling for regionalization, patient decision-making factors should be considered before any realistic implementation.

## Introduction

Over the past 40 years, the robust relationship between increased volume and improved surgical outcomes has been reinforced and supported across thousands of hospitals and dozens of different procedures [1-4]. Organizations such as the Leapfrog group and large academic centers have even advocated for the implementation of regionalizing complex procedures to high-volume centers. However, despite the abundance of evidence, the majority of complex cancer operations continue to occur at low-volume hospitals [5]. Despite the push towards regionalization, the majority of cancer patients continue to choose not to travel [6].

Finlayson and Birkmeyer found while some patients were willing to travel for a mortality benefit of 9%, 23 out of 100 patients preferred to receive their operation at a local hospital [7]. To further elucidate the factors that might motivate a patient to travel, Resio et al. asked patients to rank not only mortality and surgeon volume but also rates of complication, infection, mortality, cure, and complete resection. The authors found that 45% of patients that would be motivated for one of the mentioned factors would be resistant in terms of another factor [8]. Indeed, the decision to travel for complex cancer care is multifactorial and riddled with opposing forces. It is unknown whether patients are even aware of the volume-outcomes relationship, let alone the morbidity and mortality data. Do patients base their decision on more surgeon-related or hospital-related factors? For how long are patients willing to travel, if at all? Does that decision change if, for example, care will require complex procedures and frequent follow-up as is the case for complex cancer care? Previous surveys have focused on patients without a cancer diagnosis. Additionally, they have been conducted in regions close to quaternary institutions, neglecting the perspective of rural patients that may be significantly burdened by travel.

Our objective was to survey patients undergoing complex hepatopancreaticobiliary (HPB) cancer surgery at community hospital serving rural and metro patients. The goal of the study was to elucidate their decision-making process. We specifically focused on the following factors: awareness of the volume-outcome relationship, patient decision-making, logistical considerations of care, and burdens to travel.

## Materials and methods

Data source and setting

West Michigan Cancer Center (WMCC) Institutional Review Board approved the study. WMCC is a community oncology center that provides medical oncology, gynecology oncology, radiation oncology and surgical oncology services to the Kalamazoo-Portage metropolitan area. Kalamazoo is a medium-sized city located in Western Michigan with a population of approximately a quarter-million in the associated county. Prior to the development of the HPB program, patients in this encatchment region had to travel for HPB surgery [9]. The closest quaternary center is approximately two hours away. Inclusion criteria included all HPB surgery patients seen at the surgical oncology clinic at WMCC. Patients who were non-English speakers, possessed limited decision-making capacity or were unable to read and write were excluded from the study. A research team member administered surveys during the post-operative clinic visit within two weeks of operation. There was no incentive provided. All patients participated voluntarily and were made aware that responses would not affect the care they would receive at WMCC.

Survey development

Due to a lack of validated patient questionnaires addressing our study objectives, a questionnaire was developed to determine the following factors: awareness of the volume-outcome relationship, patient decision-making, logistical considerations of care, and burdens to travel. The survey was initially designed with 25 questions after an initial evaluation with patients, cancer providers and a review of the available literature. To characterize what patients thought about the decision of where to have surgery, we conducted two focus groups with patients who had recently undergone major surgery. We developed a draft survey instrument based on the focus groups' conclusions, input from physicians, and behavioral specialists with experience developing surveys. A cognitive interview of the completed survey with 10 patients who had undergone surgery at WMCC was done to assess for clarity, timeliness, appropriateness and that the answers were meaningful. We revised the survey on the basis of these results. Based on the initial pilot survey, the questions were modified and decreased to 18 questions. Awareness of the significance of volume in clinical outcomes was assessed by a binary response. Patient decision-making factors were assessed using a scaling format or a Likert scale and were categorized according to physician factors (doctor recommendation, confidence, family and friends recommendation), hospital-related factors and nonclinical factors [10].

Quantitative analysis

Statistical analyses were completed using the Statistical Analysis System (SAS) version 9.4 (SAS Institute Inc., Cary, NC). Survey items one and two were combined into one binary indicator for whether patients were or were not aware of operation center volume; this indicator was used for all statistical comparisons of interest. Survey responses were compared using the Chi-square test of independence, or Fisher’s exact test when assumptions were not met. P-values were adjusted using the Benjamini-Hochberg False Discovery Rate (FDR) adjustment to account for multiple testing. All adjusted p-values were compared to a significance level of α=0.05.

The amount patients were willing to travel was grouped into two groups: not willing to travel at all through willing to travel up to two hours and willing to travel two hours or more. Comparisons were made between this variable and making median income, Medicare, Medicaid, and commercial insurance, employment status, and patient race using Fisher’s exact test. P-values were adjusted using Benjamini-Hochberg FDR adjustment. Average ratings were reported for the three ranked survey questions and are reported out in ascending order (a score closer to 1 indicates greater importance, a score closer to 3 indicates less importance).

## Results

Survey response and patient characteristics

Patient volumes and clinical outcomes at our institution have been previously described [9]. A total of 76 completed surveys (89% response rate) were analyzed. 44.7% of patients had pancreas resections, and 55.3% had hepatobiliary resections. Respondents were 60.5% female, 82.9% Caucasian, 13.2% African-American and 2.6% Hispanic with a median age of 62. The majority of patients (65.7%) were under the age of 65 years, and 19.2% had a history of cancer prior to current diagnosis (Table 1). Mean time from surgery to survey completion was 17 days. There were no demographic differences between those who did and did not respond to the survey.

**Table 1:** Patient characteristics

Characteristic	No. of Respondents (%)
Age	
<45	6 (7.9)
45-59	22 (28.9)
60-74	37 (48.7)
75-90	11 (14.5)
Sex	
Male	30 (39.4)
Female	46 (60.5)
Race/ethnicity	
Caucasian	63 (82.9)
African-American	10 (13.2)
Hispanic	2 (2.6)
Asian	1 (1.3)
Annual household income, $	
<30,000	3 (3.9)
30,000 – 59,999	59 (77.6)
>60,0000	13 (17.1)
Employment Status	
Employed	30 (39.5)
Not Employed	46 (60.5)
Education Level	
Less than high school	34 (49.2)
More than high school	35 (51.7)
Insurance Status	
Medicare	36 (47.4)
Medicaid	15 (19.7)
Commercial	31 (40.8)
Marital Status	
Single	31 (43.1)
Married	41 (56.9)
Metropolitan Area Resident	
Metro	31 (40.8)
Rural	45 (59.2)
Previous Cancer Diagnosis	15 (19.2)
Surgical Procedure	
Pancreas Resections	34 (44.7)
Hepatobiliary Resections	42 (55.3)

Awareness of volume-outcome relationship

58.8% of patients were unaware of the volume-outcome relationship. There were no demographic differences in terms of race, insurance status, educational level or socioeconomic factors between those who were aware and those who were not aware of the volume-outcome relationship. In an effort to determine whether awareness of the volume-outcome relationship had a significant impact on patient preferences, a multivariable logistic regression was performed after patients were divided into either an ‘aware’ or a ‘not aware’ group. Patients who had done some research prior to surgery were more likely to be aware of the importance of volume on outcome OR 3.24 95% C.I (1.22- 8.63) p=0.0306. A significant majority of patients (89.6%) expressed a preference that physicians should be required to openly disclose the number of times they have performed that particular operation.

Patient decision-making

**Figure 1:**
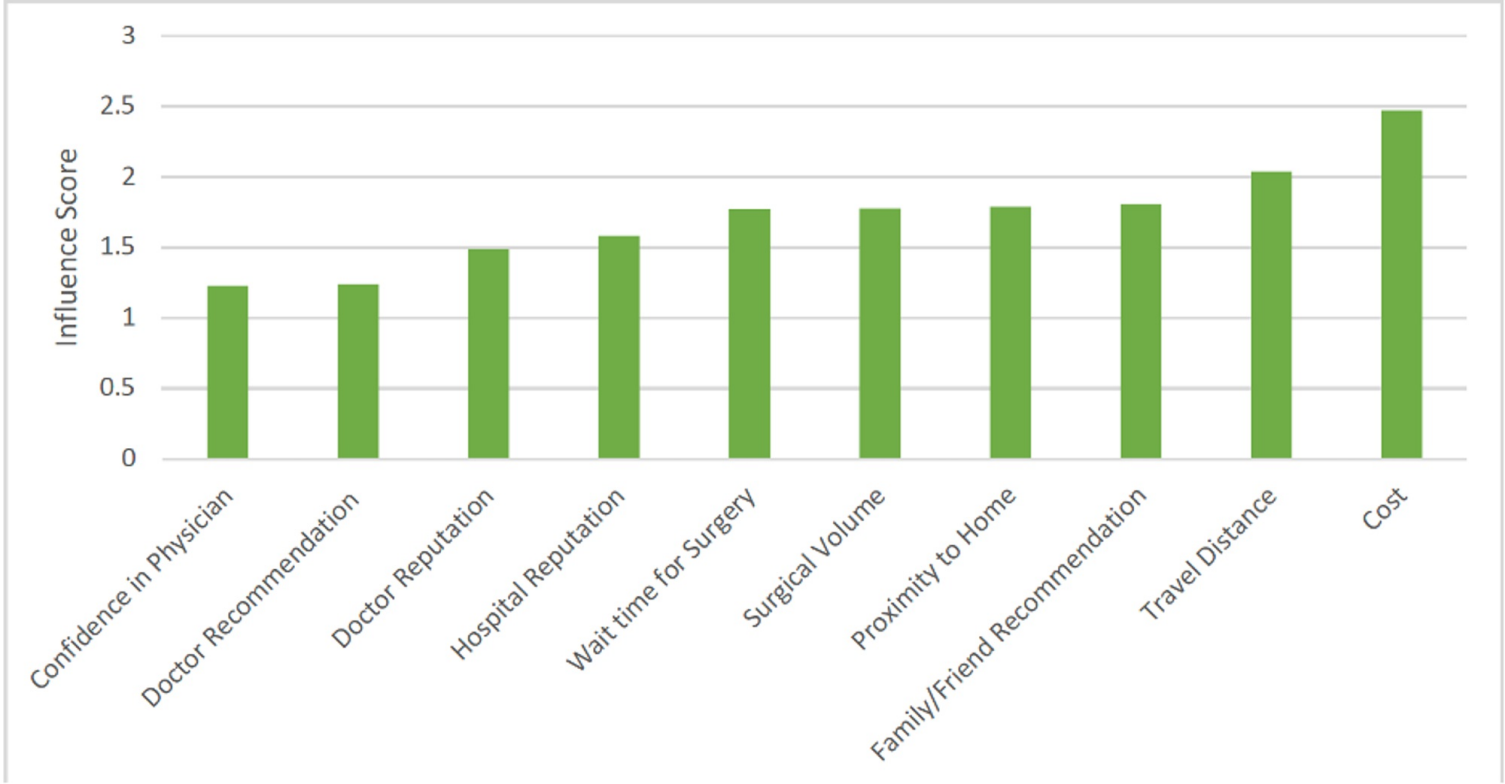
Factors affecting surgeon selection

Respondents were asked to assign a score to different factors that comprise the decision to have complex surgery, with “1” being the most important and an overall lower score representing a higher perceived impact for a particular factor. Most patients considered confidence in their physician, recommendation from their physician, and doctor’s reputation as having the most influence on their decision in selecting their surgeon (Figure 1).

**Figure 2:**
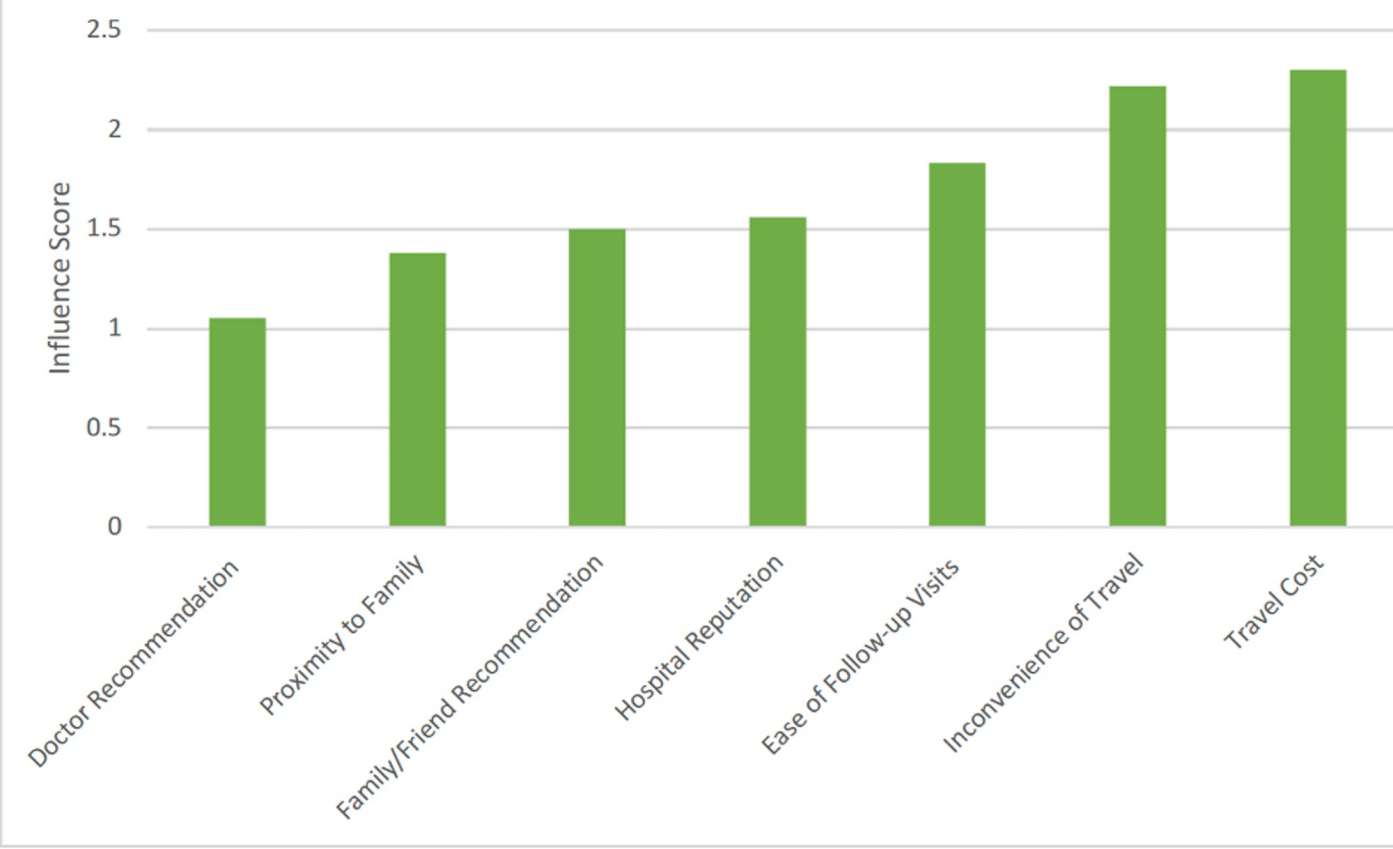
Factors affecting hospital selection

Physician factors were considered the most important factor by 64.4% of patients. Hospital related factors were considered the most important by 14.5% of patients. Respondents were asked specifically to assign an ‘influence score’ to factors regarding their surgery location. This involved assigning a numerical score to rank the ‘influence’ of various factors to characterize the relative differences in ‘influence.’ Lower scores reflect more ‘influence’ on a patient’s decision. In doing so, patients chose doctor recommendation and being ‘close to family during or after surgery’ as the most important (Figure 2).

Logistics of care

**Figure 3:**
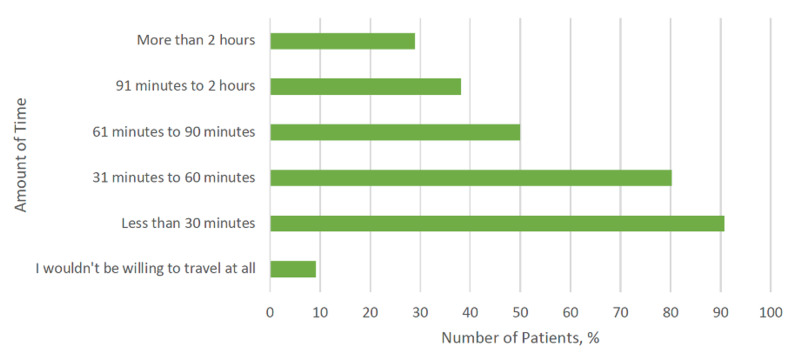
Proportion of patients willing to travel different amounts of time to receive care at a high-volume center

In an effort to further elucidate the patient’s perspective on receiving care at a high-volume center with improved outcomes, respondents were asked how long they would be willing to travel. Many patients (80.2%) indicated that they would travel up to an hour to receive complex surgery. There were also a number of patients (28.9%) that were willing to travel more than two hours to undergo complex surgery (Figure 3). To better understand how demographic characteristics might impact logistics to care, respondents were divided into two groups - those willing and those not willing to travel up to two hours. The ‘two-hour’ threshold approximates the time it would take for a patient to travel to a quaternary university program from the WMCC. There were no demographic differences between patients who were willing to travel >2 hours and those who were not willing to travel that distance. The majority of patients (61.2%) were willing to wait up to three weeks to have their operation at a hospital would be considered ‘high volume’ for a given procedure. Another group of patients (19.4%) would be willing to wait up to a month. A final minority group of patients (19.4%) would be willing to wait over a month.

**Figure 4:**
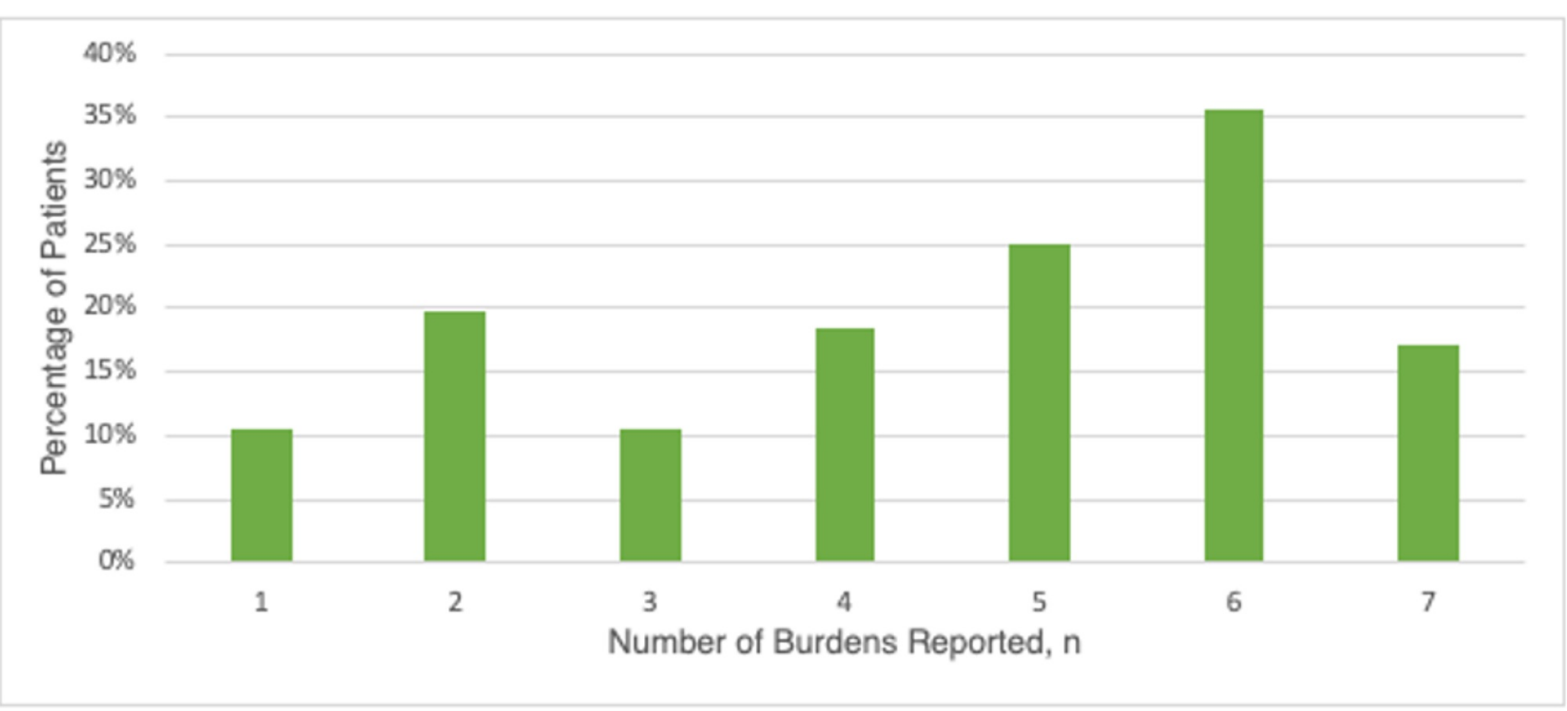
Proportion of patients reporting burdens to travel

In an attempt to characterize the burdens that a patient may encounter when traveling to receive care, patients were asked what factors had the most impact on their comfort. While 15.8% of patients claimed to have no burdens to travel, 31.6% of patients described having two or more burdens (Figure 4). A comparison between patients with three or more travel burdens did not reveal any statistically different demographic factors between the patients. The factors that most patients cited as burdens included ‘worry about not having easy access to see my doctor’ (38%), ‘inconvenience of two-hour trip’ (27%), and ‘cost of traveling’ (25%).

## Discussion

Numerous studies have documented the importance of volume in outcome for complex surgeries. This has been so widely published that it has been the subject of multiple television and national newspaper articles over the last few decades [4,11]. It is, however, unclear if this phenomenon, widely accepted by the surgical and medical community, has permeated to the general public, especially the patient who may not reside close to a tertiary academic center. Some studies have shown a trend in complex surgeries being done at high volume centers while others failed to show a sustained increase [4]. We found that in our metro, mid-sized community that the majority of respondents in our study were unaware of the volume-outcome relationship and its impact on the quality of surgery. This may explain why some authors have reported patients disregarding outcome data in selecting hospitals and sometimes bypassing high performing hospitals to get care at lower volume hospitals [6]. Our patient population is similar to multiple single institution and national representative studies of patient undergoing HPB surgery [12]. There was also no statistically significant difference in awareness of the volume-outcome relationship based on sociodemographic factors. Interestingly, even factors such as education, income level or previous history of cancer did not affect the knowledge of the importance of volume in outcomes. The 41.3% of patients who were ‘aware’ of the volume-outcomes relationship had done some research prior to their surgery (OR 3.24), suggesting that the information regarding outcomes, if sought, is readily available. However, only 50% had done some research which is consistent with other studies where the use of public performance surveys has been reported to be 20% to 56% [13,14]. Our patient population’s relatively high amount of research may contribute to their awareness of the volume-outcome relationship.

When looking at patient decision-making, awareness of volume-outcome relationship did not seem to affect what were considered the most important factors in selecting a surgeon or hospital. This suggests that, perhaps, patients are using other factors to make the decision of where to have complex surgery other than volume alone. Notably, there was no statistical difference between ‘aware’ and ‘unaware’ patients with regards to ‘time willing to wait for operation at high volume center’ or ‘time willing to travel for operation at high volume center.’ That is, those who were aware of the volume-outcome relationship were not any more likely to wait longer or travel longer to reap these purported outcome benefits associated with a higher volume center. The remarkably consistent correlation between volume and outcomes is not something most patients are aware of - and when they are, it is not the main driver in their decision-making.

Physician-specific factors had the greatest influence on a respondent’s decision in where to have complex cancer surgery. Specifically, ‘confidence’ in the surgeon had the most influence. Confidence is an abstract concept not easily quantified and comprised of a complex amalgam of recommendations from family, friends or referring physician, anecdotal experience from an in-person office visit, or perhaps even online reviews. These factors were scored as having more influence on patient decision-making than the reputation of the hospital itself, the number of times that particular surgery was performed, and even cost. This underscores the importance of the individual doctor-patient relationship and its impact on the decision of not only whether to have surgery but where to have it. The importance of physician factors has been demonstrated by other authors who have reported that factors such as confidence in the surgeon and doctor recommendation were the main determinants in their choice [10,15,16]. Volume alone may not be sufficient to ‘motivate’ a patient to travel but perhaps a physician’s reputation could be. When respondents were specifically asked about the role of location for their surgery, respondents again designated ‘doctor recommendation’ as most influential but also began designating ‘family’-based responses such as ‘being close to family’ or ‘family/friend recommendation.’ This underscores the type of factor, family and proximity to family that may be undetected in a discussion on patient outcomes focused solely on standard metrics.

A majority of respondents responded that they would travel up to an hour to receive complex surgery. However, only 49.9% would be willing to travel greater than 1 hour, which is different from a national representative survey which reported 83.1% would be willing to travel more than one hour [17]. Resio et al. national survey demonstrated that 90% of patients could be motivated to travel based on superior safety and oncologic outcomes [8]. In a Veterans Affairs (VA) study, Finlayson et al. demonstrated that patients preferred to stay at a local hospital despite a two-fold increase in mortality [18]. Similarly, 20% of patients with ovarian cancer preferred not to travel to a referral center with improved outcomes in a study performed at a quaternary institution [19]. The impact of improved outcomes was not specifically assessed in our survey. The differences in reported willingness to travel may be related to the distinct populations surveyed. The national surveys studied younger, metropolitan residents without a diagnosis of cancer or need for complex surgery and therefore their perspectives may differ from patients who are actually faced with those decisions and are from a mid-sized community. Other authors have reported socioeconomic and demographic differences in willingness to travel [16]. In our study, no demographic factors were found to impact the willingness to travel more than two hours in our study population. The heterogeneity of rural populations has been noted in the literature and emphasizes the need for studies that capture the unique needs of the rural population as a whole [20].

It stands to reason that for many patients, traveling beyond the amount they are willing can prove logistically difficult. Travel time dovetails with other logistical concerns that can make it difficult for a patient to travel to receive care. Further travel time could mean more time off work, greater transportation cost and time, increased need for childcare services, and other logistical concerns [21,22]. When patients reported potential obstacles to traveling for longer than two hours, common responses included “concern about follow up and access to physician” (36.4%), ‘inconvenience of a two-hour trip’ (26.32%), ‘the cost of traveling’ (25.0%), and ‘not having anybody to accompany them during their travel’ (18.42%). Similarly to other studies which have assessed travel burdens, 31.6% of patients in our study reported 2 or greater obstacle to travel [8].

Our study had a number of limitations. For one, only patients who had undergone complex surgery at WMCC participated in the survey. These patients had already decided on where to have their surgery and are, perhaps, the patients who had elected not to travel which therefore may bias our study findings. The perspectives of those who either chose to travel or elected not to have surgery are not reflected in our study. Our questionnaire did not specifically enumerate the potential benefit of traveling to a higher volume center, and this may have affected the patient’s responses to the survey. While our study was adequately powered, the number of respondents reflects those of a lower volume center. However, the response rate (85%) was high and represents, demographically, the general population of patients with HPB cancer residing in similarly-sized cities without proximally located high-volume centers.

## Conclusions

A majority of patients in our community are unaware of the volume-outcome relationship in complex HPB surgeries. Physician-specific factors were the most important for patients when deciding where to have surgery. The recommendation from the patient’s doctor, the perceived reputation of the surgeon, and the consequent confidence in the surgeon were the factors most influential in their decision-making. The majority of patients in our community were unwilling to travel more than \two hours and expressed financial difficulties, lack of family support and concerns with follow-up as impediments to traveling for care. Further studies could investigate the actual cost-benefit of having a cancer center in a previously deregionalized area. This study’s implications for policymakers necessitate legislating policies that are feasible and desirable for patients to adhere to. Additional efforts must be made to educate the public so that an informed decision can be made. Even more importantly, policymakers should consider the patient’s perspective on regionalization. This study would suggest patients would be better served by a policy emphasizing more centers of excellence closer to home rather than the centralization of surgical services.
